# Technically Successful Mechanical Thrombectomy for Acute Lower-Limb Ischaemia and 30-day Limb Salvage: A Single-centre Study

**DOI:** 10.1007/s00270-026-04516-1

**Published:** 2026-06-29

**Authors:** Jernej Lučev, Aleš Slanič, Marko Platnjak, Silva Breznik

**Affiliations:** Department of Radiology, University Medical Centre Maribor, Ljubljanska Ulica 5, 2000 Maribor, Slovenia

**Keywords:** Acute lower limb ischaemia, Mechanical thrombectomy, TIPI, Limb salvage, Target segment, Distal embolization, Technical success

## Abstract

**Purpose:**

To describe lesion extent, post-thrombectomy angiographic flow, and early outcome after mechanical thrombectomy for acute lower-limb ischaemia, and to assess whether angiographic technical success by Thrombo-aspiration in Peripheral Ischaemia (TIPI) grade is concordant with 30-day limb salvage.

**Materials and Methods:**

This retrospective single-centre registry study included 69 acute lower-limb mechanical thrombectomy procedures from 100 lower-limb interventions in 2019–2025. Target segments, thrombus extent, post-thrombectomy TIPI grade, and 30-day outcomes were analysed at the procedure level. Angiographic technical success was defined as TIPI grade 2–3. The primary endpoint was 30-day limb salvage. Paired discordance was assessed using the exact McNemar test.

**Results:**

Median age was 71.0 years, 25/69 limbs (36.2%) had Rutherford IIb ischaemia, and multilevel thrombosis was present in 62/69 procedures (89.9%). Tibioperoneal/tibial involvement was present in 56/69 (81.2%), and pedal arch/foot involvement in 20/69 (29.0%). Post-thrombectomy TIPI grades were 0 in 1/69, 2 in 41/69, and 3 in 27/69 procedures; angiographic technical success was achieved in 68/69 (98.6%; 95% CI, 92.2–100.0). Thirty-day limb salvage was 59/69 (85.5%; 95% CI, 75.0–92.8), with a paired gap of 13.0 percentage points. Exact McNemar testing showed discordance (*p* = 0.0039): 9 of 10 limbs not salvaged had angiographic technical success. Distal embolization was evaluable in 66 procedures and occurred in 16/66 (24.2%); 30-day mortality was 6/69 (8.7%).

**Conclusion:**

Angiographic technical success by TIPI grade 2–3 was frequent but not fully concordant with 30-day limb salvage. These findings support reporting post-thrombectomy flow and downstream perfusion rather than relying on target-segment technical success alone.

**Graphical abstract:**

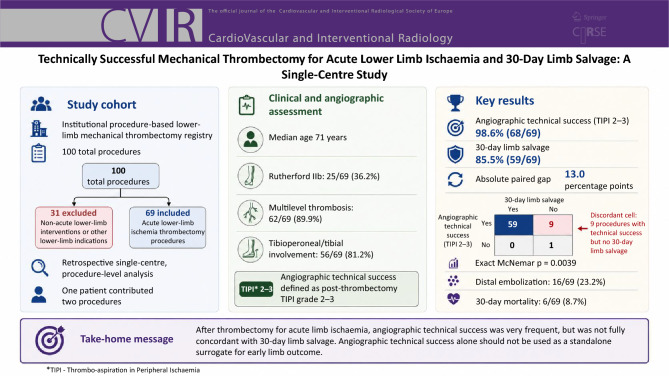

**Supplementary Information:**

The online version contains supplementary material available at 10.1007/s00270-026-04516-1.

## Introduction

Acute lower-limb ischaemia (ALI) is a vascular emergency in which timely reperfusion is required to reduce tissue loss, amputation, and death. Contemporary management increasingly includes endovascular treatment, and mechanical thrombectomy (MT) is now used in a growing proportion of patients selected for an endovascular-first approach [1–5].

However, the clinical meaning of angiographic success depends on what has been measured. Reopening of the treated arterial segment does not necessarily equate to restoration of clinically effective whole-limb perfusion. In multilevel thrombotic disease, residual distal thrombus, distal embolization, limited runoff, poor pedal filling, and tissue-level non-reperfusion may all contribute to early limb failure despite apparently successful target-segment revascularization [6–9].

To make post-thrombectomy flow assessment more reproducible, the Thrombo-aspiration in Peripheral Ischaemia (TIPI) classification has been proposed as a peripheral adaptation of TIMI-style angiographic grading, with near-complete or complete revascularization (TIPI 2–3) used as a technical success threshold in peripheral aspiration thrombectomy studies [6]. Most contemporary MT reports still emphasize technical or device-related endpoints, whereas the paired relationship between post-thrombectomy angiographic flow and early limb salvage has been less directly examined as a primary analytic question [6–12].

We therefore evaluated outcomes after mechanical thrombectomy for acute lower-limb ischaemia in a procedure-based registry cohort, incorporating target segment, level and extent of thrombosis, post-thrombectomy TIPI grade, and 30-day limb salvage. The study was designed as a non-causal test of endpoint discordance rather than a test of any specific downstream reperfusion mechanism.

## Materials and Methods

### Study Design and Setting

We performed a retrospective single-centre observational study based on a procedure level institutional lower-limb thrombectomy registry. The registry captures procedural, imaging, and outcome variables from routine clinical care. The analysed acute cohort comprised eligible procedures performed between 8 May 2019 and 7 June 2025. Because the registry was organized around index interventions, all analyses were performed at the procedure level by design rather than converted post hoc from patient-level episodes. One patient contributed two eligible procedures on opposite limbs in separate episodes. Institutional ethics approval with waiver of informed consent was obtained. This study was reported in accordance with the STROBE statement.

### Patients, Treatment, and Data Collection

**Fig. 1:**
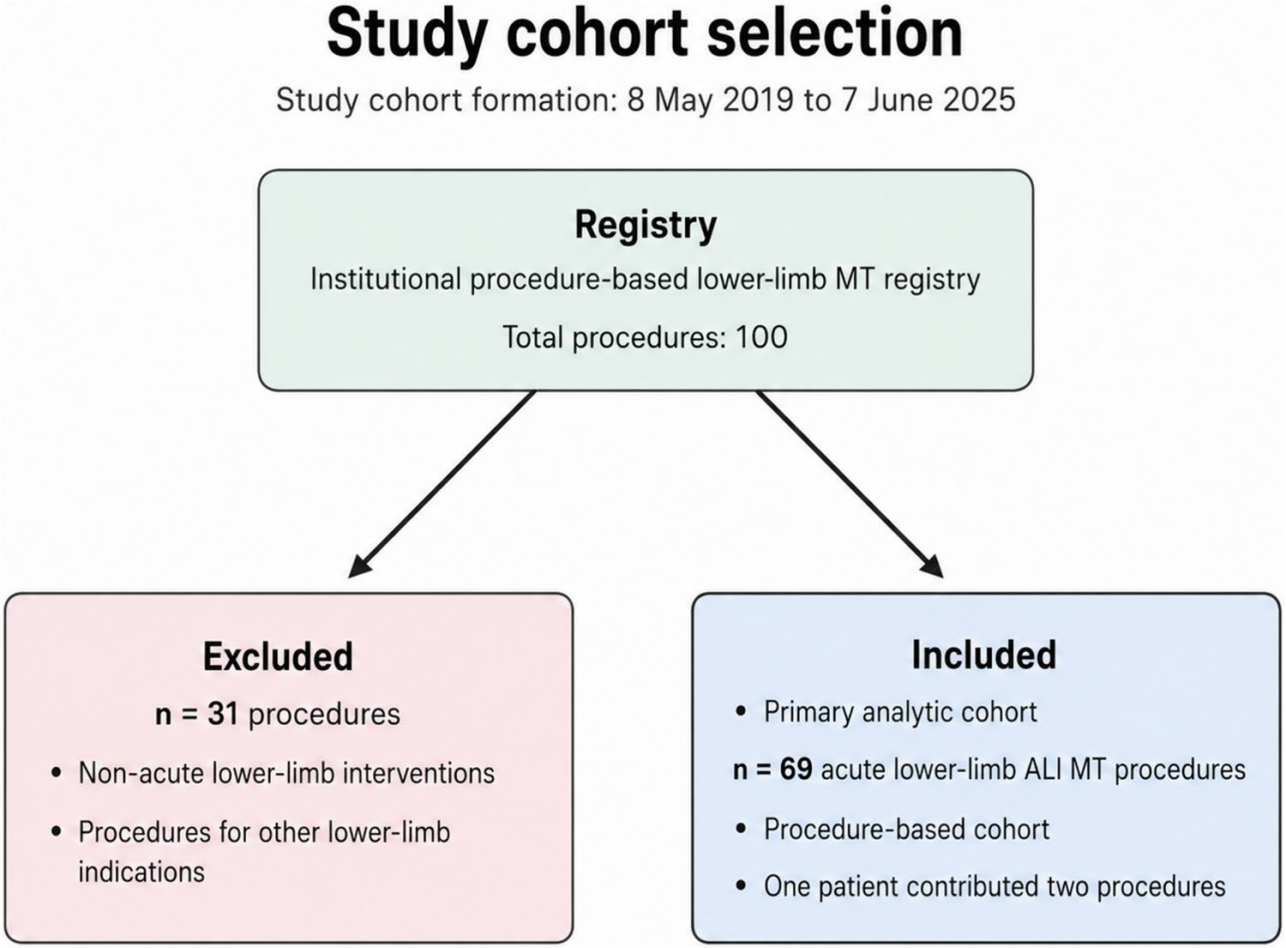
Study cohort selection. The institutional procedure-based lower-limb mechanical thrombectomy (MT) registry included 100 procedures performed between 8 May 2019 and 7 June 2025. Thirty-one procedures were excluded because they were non-acute lower-limb interventions or were performed for other lower-limb indications. The primary analytic cohort comprised 69 acute lower-limb MT procedures for acute limb ischaemia (ALI), analysed at the procedure level. One patient contributed two eligible procedures

Eligibility required acute lower-limb ischaemia with symptom duration of less than 14 days, consistent with Rutherford and ESVS definitions [1, 2]. From the institutional registry of 100 lower-limb MT procedures, 69 acute ALI procedures met eligibility for the primary analytic cohort; non-acute interventions and procedures performed for other lower-limb indications were excluded (Fig. 1). Treatment was delivered as part of routine clinical care without a predefined institutional protocol. During the retrospective study period, there was no standardized pre-procedural or completion-imaging workflow for downstream perfusion assessment, no routine dedicated foot perfusion run, and no predefined trigger for pharmacologic or catheter-based rescue manoeuvres.

Baseline variables included demographic characteristics, comorbidities, ALI aetiology, Rutherford class, symptom duration, selected laboratory parameters, target segment, level and extent of thrombosis, thrombus length, thrombus attenuation on computed tomography angiography where available, baseline TIPI grade, thrombectomy device, adjunctive endovascular treatment, local thrombolysis, post-thrombectomy TIPI grade, complications, mortality, and limb outcomes.

Mechanical thrombectomy devices were selected according to target-vessel location, thrombus distribution, and operator judgement. Rotational thrombectomy was performed with a Rotarex 6-Fr or 8-Fr catheter, depending on lesion location (BD; Becton, Dickinson and Company, Franklin Lakes, USA). Aspiration thrombectomy was performed using Eliminate 6-Fr or 8-Fr aspiration catheters (Terumo Corporation, Tokyo, Japan), Export AP aspiration catheters (Medtronic, Minneapolis, USA), or Sofia 6-Fr aspiration catheters (Terumo Corporation, Tokyo, Japan). Aspiration catheters were used with a Penumbra System aspiration pump (Penumbra, Alameda, USA) or an AXS Universal Aspiration Set aspiration pump (Stryker, Portage, USA), depending on device availability and operator preference.

Adjunctive thrombolysis, when used, consisted of post-thrombectomy intra-arterial local alteplase bolus administration through the procedural catheter at operator discretion, primarily for residual distal thrombotic burden, incomplete distal clearance, or impaired microcirculatory opening. Alteplase was administered as Actilyse Cathflo 2 mg powder for injection/infusion (Boehringer Ingelheim International GmbH, Ingelheim am Rhein, Germany), with total doses ranging from 4 to 10 mg. No patient received post-procedural catheter-directed thrombolysis, and no patient was left with an indwelling thrombolysis catheter after the procedure.

Variable completeness reflected retrospective routine-care documentation. Lactate and creatine kinase values were not prospectively mandated, were obtained selectively in urgent clinical practice, and were not systematically captured in the registry during the earlier retrospective study period. Local alteplase dose was applicable only to procedures in which adjunctive post-thrombectomy local alteplase bolus thrombolysis was administered; procedures without thrombolysis therefore had no applicable dose value rather than missing dose data.

### Imaging, Target Segments, and Procedural Assessment

Pre-procedural imaging was usually based on computed tomography angiography, although acquisition and reporting were not standardized during the retrospective study period. Target segment and thrombus extent were coded from pre-procedural imaging and procedural angiography. Segment involvement was categorized descriptively as aortoiliac, common or deep femoral, superficial femoral, popliteal or femoropopliteal bypass, tibioperoneal/tibial, and pedal arch/foot involvement. Because several procedures involved more than one arterial level, categories were not mutually exclusive. Multilevel thrombosis was defined as involvement of two or more of the broader arterial levels. Baseline distal outflow was described using the same TIPI flow framework on the pre-thrombectomy angiographic appearance. At baseline, TIPI grade 0 indicated no distal antegrade flow beyond the occluded territory, whereas TIPI grade 1 indicated minimal or incomplete antegrade distal flow. Because no procedure had near-complete or complete distal flow before thrombectomy, only baseline TIPI grades 0 and 1 were present in this cohort.

Completion angiography was performed at operator discretion and documented in the procedural report. Because TIPI grading had not been prospectively mandated during the retrospective registry period, post-thrombectomy angiographic flow was retrospectively classified by the manuscript authors according to the Thrombo-aspiration in Peripheral Ischaemia (TIPI) scale, using the final angiographic appearance after thrombectomy and any adjunctive endovascular treatment. TIPI grade 0 indicated no distal flow, grade 1 minimal or incomplete antegrade flow, grade 2 near-complete revascularization, and grade 3 complete revascularization. Consistent with previous peripheral thrombectomy reporting, TIPI grade 2 or 3 was considered angiographic technical success [6]. No blinded core-laboratory adjudication of TIPI grade was performed.

### Endpoints and Definitions

The primary clinical endpoint was 30-day limb salvage. Key secondary outcomes were angiographic technical success (TIPI 2–3), post-thrombectomy TIPI grade distribution, distal embolization, 30-day all-cause mortality, bleeding, length of hospital stay, and descriptive longer-term limb outcomes and patency where follow-up was available. Longer-term outcomes were prespecified as descriptive because follow-up ascertainment and exact event dating were incomplete in part of the retrospective registry phase.

Thirty-day limb salvage was ascertained from hospital records, follow-up visits, discharge summaries, and available external documentation. Dedicated day-30 follow-up was not mandated in this retrospective routine-care registry. Death before day 30 was counted as failure of the primary endpoint because limb salvage through day 30 could not be confirmed. When a dedicated day-30 follow-up entry was unavailable, status was adjudicated from the nearest available documentation confirming whether the limb remained in situ beyond day 30. For transparency, we additionally classified day-30 ascertainment robustness from date-stamped registry fields: 50/69 procedures had documentation confirming limb status beyond day 30, 11/69 had day-30 failure established by a dated event within 30 days (6 deaths and 5 major amputations), and 8/69 lacked such date-stamped robust ascertainment despite adjudicated endpoint status from the broader clinical record.

### Statistical Analysis

Continuous variables are reported as median and interquartile range (IQR), and categorical variables as counts, percentages, and exact 95% confidence intervals (CIs) where clinically important. Missing values were not replaced; denominators are reported transparently for each endpoint because variable completeness differed across fields. The paired comparison between angiographic technical success and 30-day limb salvage was prespecified as the central analytic test and was assessed using the exact McNemar test, because both endpoints were measured within the same procedure and the clinically relevant information lies in the discordant pairs.

We additionally report the absolute paired gap as the excess proportion of procedures in the technical success/no-limb-salvage discordant cell. Sensitivity analyses examined limb salvage among 30-day survivors, the robust day-30 ascertainment subset defined above, exclusion of one second contralateral procedure contributed by the same patient, and 30-day major amputation-free survival. Exploratory between-group comparisons used Fisher exact or Mann–Whitney U testing as appropriate, were hypothesis-generating only, and were not adjusted for multiplicity. No formal time-to-event modelling was performed because follow-up ascertainment and exact event dating were incomplete for longer-term outcomes.

## Results

### Cohort, Clinical Presentation, and Lesion Extent

The analytic cohort comprised 69 acute mechanical thrombectomy procedures. Median age was 71.0 (64.0–81.0) years, and 24/69 patients (34.8%) were women. Thrombotic ALI accounted for 34/69 procedures (49.3%). Rutherford IIa ischaemia was present in 44/69 limbs (63.8%) and Rutherford IIb in 25/69 (36.2%). Median symptom duration to puncture was 24.0 (14.0–43.0) h among 46 procedures with available data.

Occlusion was typically extensive. Median thrombus length was 185.0 (122.0–361.5) mm among 67 procedures with available measurements, and median thrombus attenuation was 57.0 (47.0–65.0) HU. Segment involvement was aortoiliac in 14/69 procedures (20.3%), common or deep femoral in 7/69 (10.1%), superficial femoral in 24/69 (34.8%), popliteal or femoropopliteal bypass in 52/69 (75.4%), tibioperoneal/tibial in 56/69 (81.2%), and pedal arch/foot in 20/69 (29.0%). Multilevel thrombosis was present in 62/69 procedures (89.9%). Additional demographic, clinical, anatomic, and procedural characteristics are summarized in Table 1. Cohort selection is summarized in Fig. 1.

**Table 1:** Baseline, lesion, and procedural characteristics

Variable	Value	Available N
Age, years	71.0 (64.0–81.0)	69
Female sex	24/69 (34.8%)	69
Current smoking	16/69 (23.2%)	69
Atrial fibrillation	23/69 (33.3%)	69
Coronary artery disease	15/69 (21.7%)	69
Diabetes mellitus	21/69 (30.4%)	69
Rutherford IIa	44/69 (63.8%)	69
Rutherford IIb	25/69 (36.2%)	69
Thrombotic ALI	34/69 (49.3%)	69
Symptom duration to puncture, h	24.0 (14.0–43.0)	46
Creatinine, µmol/L	80.0 (70.8–123.0)	68
Lactate, mmol/L	2.3 (1.5–3.1)	10
Creatine kinase, µkat/L	5.4 (1.3–12.4)	12
Thrombus length, mm	185.0 (122.0–361.5)	67
Thrombus attenuation, HU	57.0 (47.0–65.0)	69
Aortoiliac involvement	14/69 (20.3%)	69
Common/deep femoral involvement	7/69 (10.1%)	69
Superficial femoral involvement	24/69 (34.8%)	69
Popliteal or femoropopliteal bypass involvement	52/69 (75.4%)	69
Tibioperoneal/tibial involvement	56/69 (81.2%)	69
Pedal arch/foot involvement	20/69 (29.0%)	69
Multilevel thrombosis	62/69 (89.9%)	69
Baseline TIPI 0	41/69 (59.4%)	69
Baseline TIPI 1	28/69 (40.6%)	69
Aspiration thrombectomy used	48/69 (69.6%)	69
Rotarex thrombectomy used	39/69 (56.5%)	69
Combined aspiration + Rotarex	21/69 (30.4%)	69
Adjunctive local alteplase bolus thrombolysis	14/69 (20.3%)	69
Local alteplase dose, mg	4.5 (4.0–9.5) (range, 4–10)	14
Nitroglycerin rescue treatment	11/69 (15.9%)	69

Categorical variables are shown as n/N (%); continuous variables are median (interquartile range). Segment categories are not mutually exclusive because thrombosis frequently involved more than one arterial level. Laboratory and thrombus-length parameters were available only in subsets because collection during the retrospective registry phase was not fully standardized. Baseline TIPI grade describes pre-thrombectomy distal antegrade flow using the TIPI framework; only grades 0 and 1 were present before thrombectomy

### Procedural Strategies and Adjunctive Thrombolysis

Procedural strategy reflected routine operator judgement rather than a predefined treatment algorithm. Aspiration thrombectomy was used in 48/69 procedures (69.6%), Rotarex thrombectomy in 39/69 (56.5%), and a combined aspiration plus Rotarex strategy in 21/69 (30.4%). Adjunctive local thrombolysis was used in 14/69 procedures (20.3%). When used, alteplase was administered as low-dose intra-procedural local boluses, with a median dose of 4.5 (4.0–9.5) mg (range, 4–10 mg). No patient received post-procedural thrombolysis, and no patient was left with an indwelling thrombolysis catheter after the procedure. The selective and relatively low use of local thrombolysis reflected a pragmatic mechanical-first strategy in which adjunctive thrombolysis was reserved mainly for residual distal thrombotic burden, incomplete distal clearance, or impaired microcirculatory opening after thrombectomy.

### Post-Thrombectomy Flow and Early Outcomes

Post-thrombectomy TIPI grade was 0 in 1/69 procedures (1.4%), 1 in 0/69 (0.0%), 2 in 41/69 (59.4%), and 3 in 27/69 (39.1%). Angiographic technical success, defined as TIPI 2–3, was therefore achieved in 68/69 procedures (98.6%; 95% CI, 92.2–100.0).

**Fig. 2:**
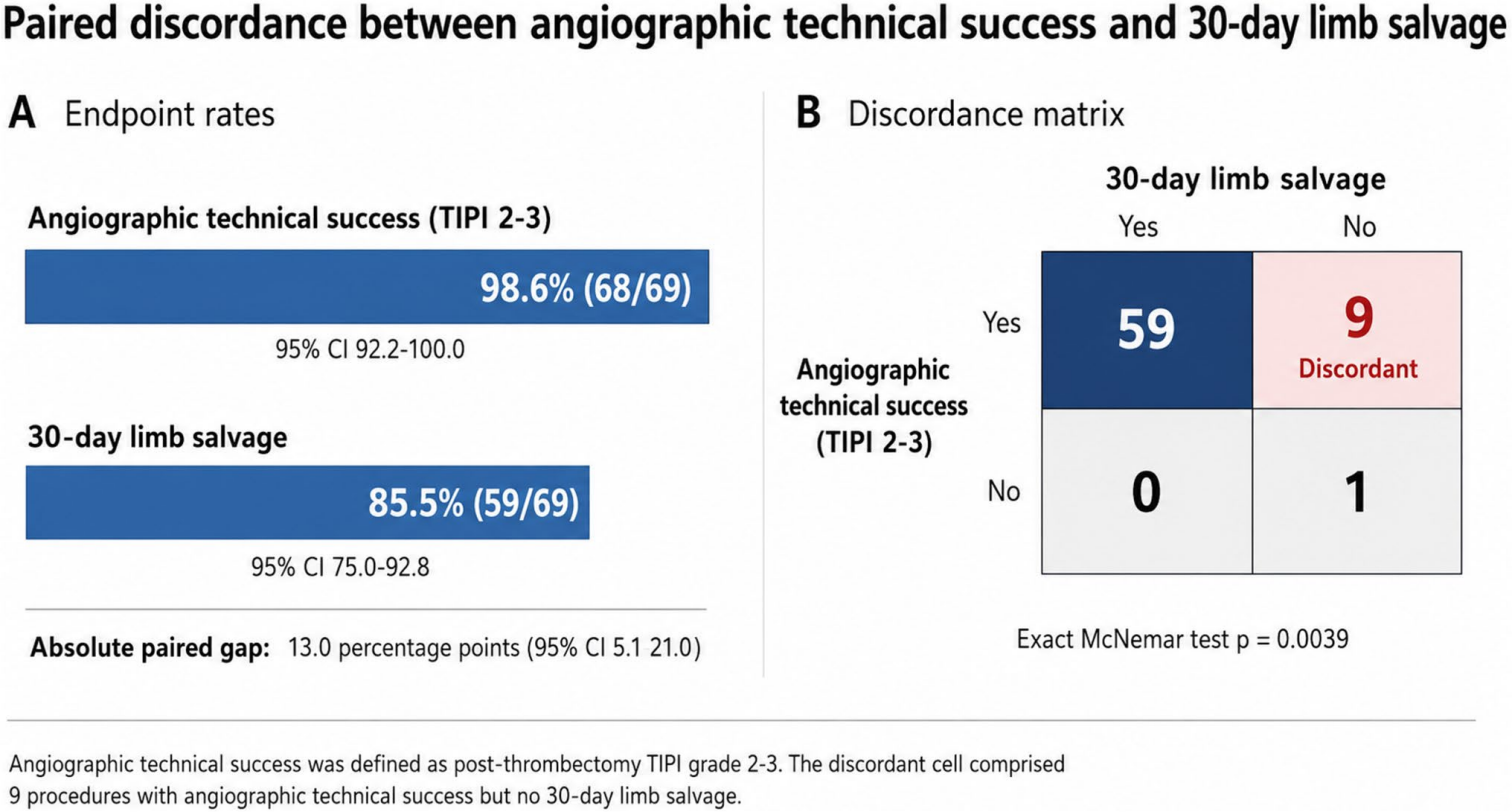
Paired discordance between angiographic technical success and 30-day limb salvage. Panel **A** shows endpoint rates for post-thrombectomy angiographic technical success, defined as TIPI grade 2–3, and 30-day limb salvage, including the absolute paired gap. Panel **B** shows the corresponding 2 × 2 paired outcome matrix used for exact McNemar testing. The discordant cell comprised nine procedures with angiographic technical success but no 30-day limb salvage. The figure is intended as a descriptive data summary rather than a mechanistic model. TIPI = Thrombo-aspiration in Peripheral Ischaemia

Early and follow-up outcomes are summarized in Table 2. Distal embolization status was evaluable in 66 procedures and was recorded in 16/66 procedures (24.2%; 95% CI, 14.5–36.4); in three procedures, the presence or absence of distal embolization could not be reliably adjudicated from available procedural/imaging documentation. The primary endpoint, 30-day limb salvage, was achieved in 59/69 procedures (85.5%; 95% CI, 75.0–92.8). The absolute paired gap between angiographic technical success and 30-day limb salvage was 13.0 percentage points (95% CI, 5.1–21.0). Exact McNemar testing demonstrated significant discordance (*p* = 0.0039): among the 10 limbs not salvaged at 30 days, technical success had nevertheless been achieved in 9. Thirty-day mortality was 6/69 (8.7%; 95% CI, 3.3–18.0). Major bleeding occurred in 3/69 procedures (4.3%), any recorded bleeding in 9/69 (13.0%), and median length of stay was 5.0 days (IQR, 3.0–9.0). The overall discordance pattern is illustrated in Fig. 2, and the paired outcome matrix is shown in Table 3.

**Table 2:** Post-thrombectomy flow and early/follow-up outcomes

Outcome	Value	Notes
Post-thrombectomy TIPI 0	1/69 (1.4%)	No distal flow
Post-thrombectomy TIPI 1	0/69 (0.0%)	Minimal/incomplete antegrade flow
Post-thrombectomy TIPI 2	41/69 (59.4%)	Near-complete revascularization
Post-thrombectomy TIPI 3	27/69 (39.1%)	Complete revascularization
Angiographic technical success	68/69 (98.6%; 95% CI, 92.2–100.0)	TIPI 2–3
Distal embolization	16/66 (24.2%; 95% CI, 14.5–36.4)	Evaluable in 66 procedures; 3 procedures excluded because status could not be reliably adjudicated from available procedural/imaging documentation
30-day limb salvage	59/69 (85.5%; 95% CI, 75.0–92.8)	Primary clinical endpoint
Absolute paired gap	13.0 percentage points (95% CI, 5.1–21.0)	Paired excess of technical success over limb salvage
30-day limb salvage among 30-day survivors	59/63 (93.7%; 95% CI, 84.5–98.2)	Sensitivity analysis
30-day limb salvage in robust ascertainment subset	51/61 (83.6%; 95% CI, 71.9–91.8)	Sensitivity analysis
30-day major amputation-free survival	60/69 (87.0%; 95% CI, 76.7–93.9)	Sensitivity analysis
90-day limb salvage	55/68 (80.9%)	Incomplete follow-up ascertainment
180-day limb salvage	53/66 (80.3%)	Incomplete follow-up ascertainment
30-day mortality	6/69 (8.7%; 95% CI, 3.3–18.0)	
6-month mortality	10/68 (14.7%)	Incomplete follow-up ascertainment
1-year mortality	10/67 (14.9%)	Incomplete follow-up ascertainment
180-day patency	30/53 (56.6%)	Among evaluable limbs
Documented amputation (any time available)	13/69 (18.8%)	11 major, 2 minor
Median time to any amputation, days	52 (10–575)	Exact dates available in 11/13 amputations
Median time to minor amputation, days	6 (range, 4–8)	2 events
Median time to major amputation, days	59 (range, 4–830)	Exact dates available in 9/11 major amputations
Major bleeding	3/69 (4.3%)	CIRSE grade > = 3
Any recorded bleeding	9/69 (13.0%)	
Median length of stay, days	5.0 (3.0–9.0)	

TIPI = Thrombo-aspiration in Peripheral Ischaemia. Angiographic technical success was defined as TIPI grade 2–3. Exact 95% confidence intervals are shown for clinically important binary endpoints. Follow-up denominators vary because outcome ascertainment was incomplete in parts of the retrospective registry phase

**Table 3:** Paired discordance between angiographic technical success and 30-day limb salvage

	30-day limb salvage: yes	30-day limb salvage: no
Angiographic technical success (TIPI 2–3): yes	59	9
Angiographic technical success (TIPI 2–3): no	0	1

Exact McNemar test for paired discordance: *p* = 0.0039. The absolute paired gap was 13.0 percentage points (95% CI, 5.1–21.0). This paired test was used because both endpoints were measured within the same procedure and the inferential question concerned discordant pairs rather than comparison of independent proportions

### Sensitivity and Longer-term Descriptive Outcomes

Sensitivity analyses were directionally consistent with the primary finding. Among 30-day survivors, limb salvage was 59/63 (93.7%; 95% CI, 84.5–98.2); the paired discordance signal was attenuated because three of the four remaining failures still had angiographic technical success (exact McNemar *p* = 0.25). In the robust day-30 ascertainment subset defined by dated events or documentation beyond day 30, limb salvage was 51/61 (83.6%; 95% CI, 71.9–91.8), and the main discordance pattern was unchanged (exact McNemar *p* = 0.0039). After exclusion of the second contralateral procedure contributed by the same patient, results were materially unchanged. Using 30-day major amputation-free survival as an alternative endpoint, success was 60/69 (87.0%; 95% CI, 76.7–93.9), again lower than angiographic technical success.

Longer-term outcomes are reported for completeness but should be interpreted descriptively because follow-up ascertainment was incomplete and exact event dates were not consistently available. Limb salvage was 55/68 (80.9%) at 90 days and 53/66 (80.3%) at 180 days. Mortality was 10/68 (14.7%) at 6 months and 10/67 (14.9%) at 1 year. Patency at 180 days was documented in 30/53 evaluable limbs (56.6%). Across available follow-up, 13/69 procedures (18.8%) were associated with a documented amputation, including 11 major and 2 minor amputations. Exact amputation dates were available in 11/13 procedures; among these, median time from MT to any amputation was 52 days (IQR, 10–575).

### Exploratory Comparisons

Exploratory comparisons are provided as descriptive context only and should not be interpreted as inferential evidence. Procedures without 30-day limb salvage more often involved older patients, Rutherford IIb ischaemia, and pedal arch or foot involvement. No statistically robust differences were identified for thrombus length, thrombus attenuation, anatomic level, distal embolization, Rotarex use, or adjunctive local thrombolysis. Detailed exploratory comparisons are shown in Table 4, and descriptive characteristics of the nine discordant procedures are summarized in Online Resource 1.

**Table 4:** Exploratory clinical, anatomical, and procedural characteristics by 30-day limb salvage status

Variable	30-day limb salvage (N = 59)	No 30-day limb salvage (N = 10)	Exploratory p value
Age, years	70.0 (62.5–80.5) [N = 59]	79.5 (66.2–87.5) [N = 10]	0.170
Symptom duration to puncture, h	24.0 (14.0–42.5) [N = 39]	29.0 (18.0–62.5) [N = 7]	0.351
Thrombus length, mm	185.0 (123.0–350.0) [N = 57]	195.5 (113.5–392.2) [N = 10]	0.944
Thrombus attenuation, HU	56.0 (46.0–65.0) [N = 59]	58.0 (53.0–67.0) [N = 10]	0.551
Rutherford IIb	19/59 (32.2%)	6/10 (60.0%)	0.152
Aortoiliac involvement	11/59 (18.6%)	3/10 (30.0%)	0.412
Femoropopliteal involvement	52/59 (88.1%)	9/10 (90.0%)	1.000
Tibial involvement	49/59 (83.1%)	7/10 (70.0%)	0.385
Pedal arch/foot involvement	15/59 (25.4%)	5/10 (50.0%)	0.140
Multilevel thrombosis	53/59 (89.8%)	9/10 (90.0%)	1.000
TIPI grade 3 after thrombectomy	24/59 (40.7%)	3/10 (30.0%)	0.729
Distal embolization	14/56 (25.0%) [N = 56]	2/10 (20.0%) [N = 10]	1.000
Adjunctive local thrombolysis	11/59 (18.6%)	3/10 (30.0%)	0.412
Rotarex used	35/59 (59.3%)	4/10 (40.0%)	0.312

These comparisons are strictly exploratory and descriptive; p values are shown only to orient the reader and must not be interpreted as inferential evidence. Continuous variables are shown as median (interquartile range); N is shown where data availability differed between groups. For distal embolization, denominators reflect evaluable procedures only

## Discussion

In this retrospective single-centre procedure-based cohort, mechanical thrombectomy for acute lower-limb ischaemia achieved very high angiographic technical success when assessed by TIPI grade 2–3, yet 30-day limb salvage was meaningfully lower. The key finding was paired endpoint discordance: technical success had been achieved in 9 of the 10 limbs not salvaged at 30 days, corresponding to a 13.0 percentage-point gap between angiographic technical success and early limb salvage. The study should therefore be read as demonstrating paired endpoint discordance, not as proving that technical success causes or fails to cause limb salvage. This central finding is visually summarized in Fig. 2.

The cohort was clinically complex: more than one-third of limbs presented with Rutherford IIb ischaemia, thrombus length was substantial, and nearly 90% of procedures involved multilevel thrombosis. Tibioperoneal/tibial involvement was frequent and pedal arch or foot involvement was present in almost one-third of procedures. This pattern is important because mechanical clearance of the main treated segment may not fully capture distal runoff, pedal filling, and tissue-level reperfusion, particularly when thrombosis extends below the knee or into the foot.

In the present cohort, nearly all procedures achieved TIPI 2 or 3, confirming near-complete or complete angiographic revascularization of the treated territory in most procedures. However, the lower 30-day limb-salvage rate demonstrates that even a standardized angiographic flow endpoint cannot be used as a complete substitute for patient-relevant early outcome. The practical message is not that angiographic technical success is unimportant, but that it should be reported together with clinical outcome and downstream perfusion descriptors.

These findings should be interpreted within the context of contemporary ALI thrombectomy literature, which generally reports high technical success but substantial heterogeneity in patient and lesion mix, endpoint definitions, and follow-up structure [6–12]. In that context, the present cohort is not primarily informative because of device-specific performance, but because it explicitly tests whether a commonly emphasized procedural endpoint tracks adequately with an early patient-relevant outcome. Precisely because the technical endpoint was conventional and pragmatic, testing it against 30-day limb salvage is methodologically relevant.

The relatively selective use of adjunctive local thrombolysis also reflects routine practice in an elderly and comorbid ALI population. Prolonged catheter-directed thrombolysis may be useful in selected patients, but it can delay immediate revascularization and carries bleeding risk. In this cohort, thrombolysis was not used as a primary prolonged infusion strategy; instead, small post-thrombectomy intra-arterial local alteplase boluses were used selectively during the procedure when residual distal thrombotic burden, incomplete distal clearance, or impaired microcirculatory opening was encountered. No post-procedural catheter-directed thrombolysis was performed. This approach is experience-based, but it is consistent with the broader rationale of minimizing thrombolytic exposure while using mechanical thrombectomy for rapid thrombus debulking. Published data also support the concept that low-dose thrombolysis protocols can reduce major bleeding compared with higher-dose strategies while maintaining clinically acceptable effectiveness in selected lower-extremity arterial occlusions [1, 13].

From a practical interventional standpoint, the findings support a broader completion-assessment framework after ALI thrombectomy than target-segment technical success alone. Where feasible, future prospective workflows should standardize documentation of treated-segment

 patency, TIPI grade, runoff restoration, pedal filling, residual distal filling defects, distal embolization, and any rescue manoeuvres required to address downstream perfusion compromise. Such reporting would improve both immediate procedural decision-making and the interpretability of endpoint-based ALI thrombectomy studies.

This study has important limitations. It was retrospective, single-centre, and modest in size, with only 10 failures of the primary endpoint, limiting statistical depth and precluding robust multivariable modelling. The analysis was procedure-based, although the only duplicated patient contributed two temporally separate contralateral procedures and sensitivity analysis excluding one of them was unchanged. Variable completeness was limited for some retrospectively extracted baseline laboratory variables, particularly lactate and creatine kinase, because these tests were obtained selectively during urgent routine care and were not prospectively mandated or systematically captured in the registry. Imaging acquisition, completion angiography, adjunctive treatment, and rescue escalation were not standardized. TIPI grade was assigned from completion angiographic information available in the registry rather than by a blinded core laboratory, and distal embolization was not independently adjudicated. Thirty-day limb-salvage ascertainment was partly adjudicated from nearest available documentation rather than uniform scheduled day-30 visits, but the misclassification concern was directly tested and the main signal persisted in the robust ascertainment subset. Longer-term follow-up was incomplete for several descriptive endpoints, and exact event dates were not consistently available for time-to-event analysis. Accordingly, the study should be interpreted as a signal-generating demonstration of clinically relevant paired endpoint discordance and of the need for better downstream perfusion assessment, rather than as proof of a specific downstream reperfusion mechanism.

## Conclusion

In this retrospective single-centre registry cohort of acute lower-limb ischaemia thrombectomy procedures, angiographic technical success defined as TIPI grade 2–3 was achieved in almost all procedures, but 30-day limb salvage was lower. Significant paired endpoint discordance indicates that angiographic technical success is insufficient as a standalone surrogate for early limb outcome. Standardized reporting of target segment, thrombus extent, post-thrombectomy flow, runoff, pedal filling, and clinical limb outcomes should be incorporated into future prospective ALI thrombectomy studies.

## Supplementary Information

Below is the link to the electronic supplementary material.

Supplementary file1 (DOCX 29 kb)

## Data Availability

The datasets generated and/or analysed during the current study are available from the corresponding author on reasonable request.
