# Supplementary material for: Technically Successful Mechanical Thrombectomy for Acute Lower-Limb Ischaemia and 30-day Limb Salvage: A Single-centre Study
Source: Cardiovasc Intervent Radiol. 2026 Jun 29;49(8):1479–87. doi: 10.1007/s00270-026-04516-1 (PMC13429546; doi:10.1007/s00270-026-04516-1)
Supplement: Supplementary file 1 — Supplementary file1 (DOCX 29 kb) [file 270_2026_4516_MOESM1_ESM.docx]

**Online Resource 1**

# Descriptive characteristics of the nine discordant procedures with angiographic technical success (TIPI 2-3) but no 30-day limb salvage.

This supplementary table is descriptive only and is intended to characterize the discordant phenotype. It expands the case summaries by clarifying whether failure was driven by major amputation, death, or non-salvage documented from follow-up records. Distal embolization was not available as a consistently structured case-level variable in the retrospective registry spreadsheet and is therefore not tabulated here.

## Online Resource 1A. Baseline and anatomic descriptors.

| Procedure ID | Age, years | Rutherford class | Tibial involvement | Pedal/foot involvement |
| --- | --- | --- | --- | --- |
| 3 | 86 | IIa | No | No |
| 5 | 50 | IIb | Yes | Yes |
| 39 | 66 | IIb | No | No |
| 46 | 81 | IIa | Yes | Yes |
| 53 | 78 | IIb | No | No |
| 54 | 66 | IIa | Yes | Yes |
| 69 | 89 | IIb | Yes | No |
| 84 | 67 | IIa | Yes | Yes |
| 94 | 104 | IIb | Yes | No |

## Online Resource 1B. Adjunctive treatment and outcome descriptors.

| Procedure ID | Adjunctive local thrombolysis | Nitroglycerin | Verapamil | Major bleeding | 30-day failure mode | Ascertainment anchor | Days to amputation |
| --- | --- | --- | --- | --- | --- | --- | --- |
| 3 | No | No | No | No | Major amputation | amp ≤ 30 | 19 |
| 5 | Yes | No | No | No | Major amputation | amp ≤ 30 | 4 |
| 39 | No | No | No | No | Death | death ≤ 30 | — |
| 46 | No | No | No | No | Non-salvage without dated death/amputation | last ≥ 30 | — |
| 53 | No | No | No | No | Death | death ≤ 30 | — |
| 54 | No | No | No | Yes | Death | death ≤ 30 | — |
| 69 | No | No | No | No | Death | death ≤ 30 | — |
| 84 | Yes | Yes | No | No | Death | death ≤ 30 | — |
| 94 | No | No | No | No | Death | death ≤ 30 | — |

Note. The table summarizes the nine procedures that populated the clinically relevant discordant cell of Table 3, i.e., angiographic technical success (TIPI 2-3)with no 30-day limb salvage. Failure modes included two early major amputations, six deaths without documented major amputation within 30 days, and one non-salvage case adjudicated from follow-up documentation without a dated death or amputation event. The table is descriptive only and should not be interpreted as inferential evidence.
